# *Helicobacter pylori* Infection in Children Versus Adults, Differences in Management Guidelines: Risks and Benefits of Treatment in Childhood

**DOI:** 10.1111/hel.70063

**Published:** 2025-08-12

**Authors:** Matjaž Homan, Zrinjka Mišak, Francis Megraud, Michal Kori

**Affiliations:** ^1^ Department of Gastroenterology, Hepatology, and Nutrition University Children's Hospital, Faculty of Medicine, University of Ljubljana Ljubljana Slovenia; ^2^ Referral Center for Pediatric Gastroenterology and Nutrition, Children's Hospital Zagreb, School of Medicine, University of Zagreb Zagreb Croatia; ^3^ INSERM U1312 BRIC, University of Bordeaux Bordeaux France; ^4^ Pediatric Gastroenterology, Kaplan Medical Center, Faculty of Medicine Hebrew University Jerusalem Israel

**Keywords:** benefits, children, eradication, *Helicobacter pylori*, risks

## Abstract

*Helicobacter pylori* infection in children and adults differs in several aspects such as the natural history, prevalence, the clinical presentations and complications, antibiotic resistance rates, treatment options, and the success rates of treatment. Due to all the abovementioned differences, management guidelines and recommendations are different between children and adults. In parallel to the steady decrease in the rate of *H. pylori* infection in the Western world in recent years, both in children and adults, antibiotic resistance rates have risen to alarming rates. The risk and benefits of eradication treatment, especially in children, must be considered when deciding “to treat or not.” The risks include the negative effects of antibiotics, treatment failure, and reinfection as well as the possibility of losing the “protective effect” of *H. pylori* on atopy, allergy, and possibly on other gastrointestinal diseases. On the other hand, there are also many benefits of eradication therapy such as prevention of gastric complication and associated non‐gastric complications as well as reduction of parental anxiety of nontreatment. This review summarizes the differences related to *H. pylori* in children versus adults and the risks and benefits of treatment in children.

## 1 Introduction

*Helicobacter pylori* remains a major health problem worldwide, causing considerable morbidity and mortality due to peptic ulcer disease (PUD) and gastric cancer (GC), mainly in adults [1]. The discovery of *H. pylori* in 1982 and later the recognition of *H. pylori* as the cause of the majority of duodenal and gastric ulcers was a seminal medical breakthrough, leading to granting Marshall and Warren the Nobel Prize in Medicine in 2005 [2]. Even though more than 40 years have passed since the discovery of *H. pylori* , and although the incidence of the infection is decreasing, especially in industrialized countries, the bacterium still infects over half of the world's population and is a major health concern [3, 4]. The optimal management and treatment options remain unsettled and are evolving with increasing antimicrobial resistance. Despite decades of clinical practice and research in the field, major controversies and challenges remain regarding the decision of who to treat and which are the best treatment options.

Management guidelines differ significantly between children and adults, but not only because “children are not just small adults.” The natural history, the prevalence of infection, the clinical presentations, and complications are different between children and adults, as well as the risks and benefits of treatment in different age groups and areas of the world. The adult guidelines recommend treating all infected people, especially due to the risk of GC [1]. Conversely, in the pediatric guidelines, treatment recommendations are restricted to limited situations [5]. The most recent updated guidelines for children recommend treatment only in children with peptic erosions or ulcers, in children with iron deficiency anemia unresponsive to iron supplementation, and in children with first‐degree relatives positive for GC.

### 1.1 The Acquisition and Prevalence of *H. pylori* Infection

*H. pylori* infection is acquired in early childhood, usually below 5 years of age in both high and low prevalence countries [6]. The major determinant of the prevalence of infection is the socioeconomic status in childhood, including hygiene, sanitation, density of living, and educational level. Infection usually persists for life unless treated with an appropriate eradication protocol. Transient infection may occur in some infants. In adults, spontaneous eradication may occur when a longstanding infection causes widespread gastric mucosal atrophy and metaplasia with achlorhydria. Two meta‐analyses, which studied the worldwide prevalence of *H. pylori* infection and included children, showed a seroprevalence of 32.6% (95% CI: 28.4–36.8) and 33% (95% CI: 27%–38%) in pediatric patients compared to more than 50% in adults [7, 8]. Moreover, the latest data on global prevalence showed a downward trend in adults to 43.7%, but not in children where the numbers are still as high as 34.4% [9].

### 1.2 The Consequences of *H. pylori* Infection—Adults Versus Children

The consequences of infection differ significantly between children and adults.

In adults, *H. pylori* infection may be associated with dyspepsia even in the absence of PUD. However, most of the infected adults (80%) remain asymptomatic. Since the Kyoto Consensus, most of the guidelines, including the VIth Maastricht guidelines and the recent Taipei Global Consensus II, recommend eradicating *H. pylori* infection in any situation [1, 10, 11]. It is also advised to implement screen‐and‐treat programs to eradicate *H. pylori* to prevent GC in countries with a high prevalence of the infection, and for countries with a low or middle prevalence of *H. pylori* infection, to target only the population groups with a high prevalence, for example, the migrant populations. Currently, the EU is sponsoring trials to determine the best conditions to reach this goal in terms of age, diagnostic tests, and treatment regimens (TOGAS, Eurohelican) [12]. Although the benefit of *H. pylori* eradication is obvious, it must be weighed against the possible negative impact on the patient because all treatments lead to adverse events, and so the presence of comorbidities must be considered.

In children, the infection is almost always asymptomatic, with 95% of the children being without any symptoms due to *H. pylori* infection. In adults, only a third or even less of infected patients with disorders of gut–brain interaction (functional dyspepsia) will have sustained relief of symptoms after eradication therapy [13, 14]. In pediatric patients, epidemiological studies report no difference in the prevalence of recurrent abdominal symptoms between those who are infected with *H. pylori* and those who are not [15, 16]. PUD will develop in up to 5%–15% of infected adults over time, with a possible risk of gastrointestinal bleeding. In pediatric infected patients, the results of the EuroPedHP Registry (2013 to 2016) demonstrated a much lower rate of gastric/duodenal ulcers or erosions, which were found in 5.1% and 12.8%, respectively [17]. Longstanding active *H. pylori* gastritis leads to gastric mucosal atrophy and intestinal metaplasia. In the minority of adult patients, premalignant mucosal conditions progress to dysplasia (clinically silent) which may progress to non‐cardia GC. The progression occurs over many years, and these lesions are very rare in childhood [18]. Two recent pediatric studies demonstrated a potential reversibility of the rare gastric intestinal metaplasia in pediatric patients [19, 20].

The individual lifetime risk of GC in *H. pylori* ‐infected people is 1.5%–2%. The rate of GC differs among populations, with countries in East Asia, Eastern and Southern Europe, and South America having a much higher incidence of non‐cardia GC than North and West European countries. However, the impact of *H. pylori* –associated GC remains substantial, with over 1,000,000 new cases worldwide and 800,000 deaths in 2020. The number needed to treat *H. pylori* infection to prevent one GC onset was 45 (95% CI: 35–74) while the number needed to treat to prevent one death from GC was 92.5 (95% CI: 58–629) [21]. A family history of GC in a first‐degree relative is associated with a two‐ to threefold increased risk of developing this disease. Thus, both adult and pediatric guidelines recommend treating first‐degree relatives of GC patients. Gastric mucosa‐associated lymphoid tissue (MALT) lymphoma is another rare consequence of *H. pylori* infection, which can regress and may be cured with *H. pylori* eradication [22]. MALT lymphoma has been diagnosed in pediatric patients; however, its occurrence is extremely rare. In the last decade, no new reports regarding MALT lymphoma in childhood were published; therefore, in the updated guidelines, the problem is no longer addressed.

### 1.3 Risks of *H. pylori* Eradication in Children and Adults

The adult guidelines recommend eradication in all infected patients, whereas pediatric guidelines are more restrictive [1, 5]. The eradication in children is indicated only under specific conditions because treating *H. pylori,* especially in young children, can have some negative consequences specific to this age group, such as losing the protective/positive role in several extra‐gastric diseases, especially in allergic disorders. The risk of reinfection is also higher in childhood, as well as the risk of changing the microbiota which is being established at this period of life.

Other consequences, for example, the risk of treatment failure and the emergence of resistance in *H. pylori* and other gut bacteria, are common to children and adults.

#### 1.3.1 Loss of “Protection” of *H. pylori* Against Immune/Allergic Mediated Disease

Atopic diseases, allergy, and asthma are multifactorial diseases, and infection with *H. pylori* can be a protective factor. The best evidence for protection we have is for asthma. In general, several studies confirmed an inverse relationship between asthma and *H. pylori* infection. Moreover, asthma risk can be lower in children infected with CagA‐positive *H. pylori* [23]. The possible protective role of CagA‐positive *H. pylori* infection was confirmed in a recent meta‐analysis, including 18 studies with more than 17,000 children [24]. There are several hypotheses regarding possible mechanisms protecting against asthma. The “hygiene hypothesis” suggests that exposure in early childhood to certain infectious agents may improve the maturation of the immune system. In the developed world, a lack of exposure to various microorganisms can cause defective immune tolerance, leading to a higher susceptibility to allergic diseases, including asthma. Moreover, the composition of gut microbiota affects immune development. *H. pylori* colonizing the stomach can affect intestinal microbiota by changing gastric acid levels, promoting the growth of acid‐sensitive bacteria in the intestine [25]. In addition, the microorganisms, including *H. pylori,* can shift the Th1/Th2 balance and promote Th1 response, which is protective against the development of allergic asthma [26]. *H. pylori* is also able to promote transformation of dendritic cells into tolerogenic dendritic cells, which convert naive T cells into FoxP3 + Treg with high suppressive activity against airway inflammation [27].

*H. pylori* has also been implicated as a potential contributing factor for other conditions such as obesity, inflammatory bowel disease, eosinophilic disease, and celiac disease.

The studies and meta‐analysis showed inverse correlations for abovementioned conditions; however, studies showing causality are still lacking [28, 29, 30, 31].

#### 1.3.2 Risks of Re‐Infection

After successful eradication, the recurrence of infection can occur by recrudescence or reinfection. Compared with reinfection, the time window for recrudescence is shorter and lasts maximally for one year after eradication. The recurrence rate is higher in children than in adults. In children, the recrudescence and reinfection rates were 6% and 10%, whereas in adults, they were 2.2% and 3.1%, respectively [3]. Moreover, according to the global data published in 2024, the annual *H. pylori* recurrence rates in children aged ≤ 5 years, ≤ 10 years, and 11–18 years were 30%, 14%, and 8%, respectively [32]. The recurrence rate is inversely related to the human development index and directly related to the prevalence of *H. pylori* infection in the country. Therefore, in high‐prevalence countries like China, studies are ongoing with a family‐based *H. pylori* prevention and eradication strategy to prevent transmission in families [33].

#### 1.3.3 Risk of Change of the Gut Microbiota

Regardless of the type of eradication protocol used, the gut microbiota is changed to some extent. Normally, *Bacteroides* and *Firmicutes* constitute more than 90% of the gut microbiota, whereas in children after eradication, these phyla are partly replaced with *Proteobacteria* [34]. It takes at least one year for microbiota dysbiosis to return to the pre‐eradication level. The extent and speed of the microbiota restoration differ between studies [35]. In addition, studies addressing the long‐term effects of *H. pylori* eradication on gut microbiota are limited.

#### 1.3.4 Risks of Emergence of Resistant Strains

It is mandatory that patients are treated for infectious diseases with the most efficient antibiotics to prevent the increasing rate of antibiotic resistance worldwide. According to the latest *H. pylori* guidelines for children, a minimum of two antibiotics is necessary for at least 10 days in quadruple therapy, but mostly even for 14 days, to successfully eradicate *H. pylori* in the stomach [5]. With prolonged double antibiotic therapy, children are frequently noncompliant, mostly due to adverse events, the most important cause of unsuccessful eradication therapy. In addition, noncompliance increases the risk of inducing antibiotic resistance of *H. pylori* strains [36]. Boyanova et al. in a recent publication investigated resistance in Bulgaria and concluded not only that resistance rates of *H. pylori* strains are overall high, but also that the heteroresistance rate was 19.8% (out of 106 strains infecting children), including double, triple, and even quadruple resistance in 13.2%, 5.7%, and 0.9%, respectively [37].

#### 1.3.5 Risks of Treatment Failure

**FIGURE 1:**
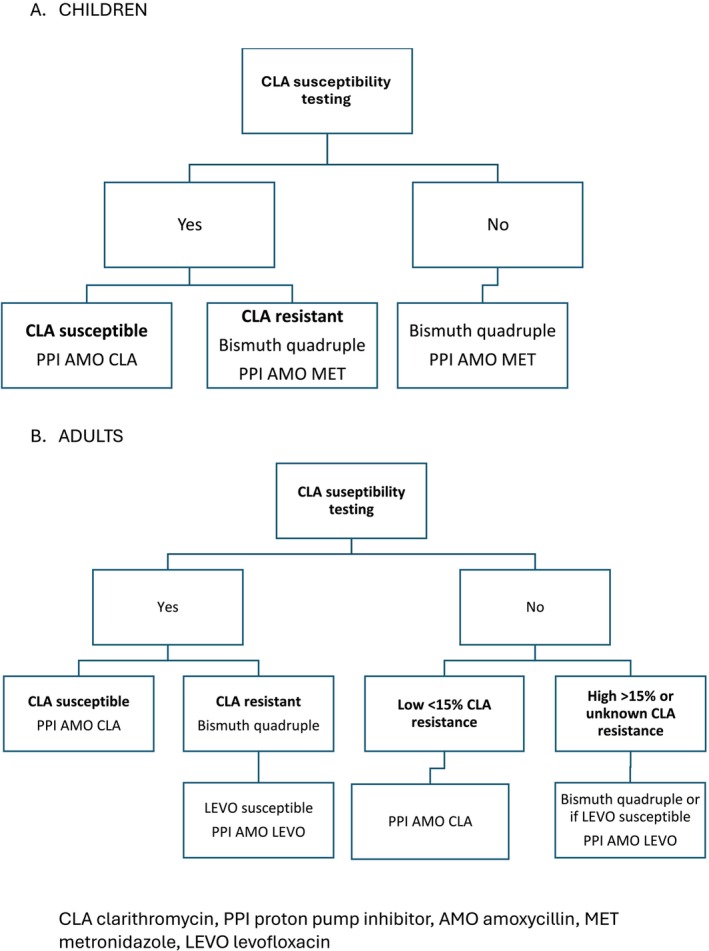
First‐line treatment: Children versus adults.

Antibiotic resistance for *H. pylori* infection is a worldwide phenomenon. Unfortunately, the resistance, in general, is higher than 15% for both of the most used antibiotics, clarithromycin and metronidazole [38]. Therefore, in line with the latest pediatric guidelines, the therapy should be prescribed after obtaining the results of antibiotic susceptibility testing (AST) based on cultivation or molecular biopsy‐based test (Figure 1) [5, 39]. If the data concerning clarithromycin resistance are not available or the strain is resistant to clarithromycin, bismuth‐based quadruple therapy should be prescribed. Without AST, the overall eradication rate is around 70%; but even if the eradication protocol is prescribed according to the results of *H. pylori* AST, the eradication success may not reach the desirable 90% [40, 41] (Table 1).

**TABLE 1:** Differences in treatment indications between children and adults.

children	adults
**Absolute treatment indications**	**Absolute treatment indications**
Duodenal and/or gastric ulcer, erosions, with or without complications	Past or present duodenal and/or gastric ulcer, with or without complications
Iron‐deficiency anemia unresponsive to iron supplementation	Unexplained iron‐deficiency anemia
Patients who have first‐degree relatives with gastric cancer	Patients who have first‐degree relatives with gastric cancer
Gastric mucosa‐associated lymphoid tissue (MALT) lymphoma^a^	Gastric mucosa‐associated lymphoid tissue (MALT) lymphoma
**Pediatric treatment dilemmas**	Indications only in adults
Nodular gastritis was found during endoscopy performed for abdominal pain	Following resection of early gastric cancer
*H. pylori* as an incidental finding during endoscopy performed to investigate other gastrointestinal diseases (inflammatory bowel disease, celiac disease, eosinophilic esophagitis)	Gastric mucosal atrophy or intestinal metaplasia
Treatment not recommended in children	Treatment recommended in adults
Disorders of gut‐brain interaction (functional dyspepsia)	Disorders of gut‐brain interaction (functional dyspepsia)
Idiopathic thrombocytopenic purpura	Idiopathic thrombocytopenic purpura
As a strategy for gastric cancer prevention in communities with high incidence	As a strategy for gastric cancer prevention in communities with high incidence
Not addressed in the pediatric guidelines	Treatment recommended in adults
Patients' wishes	Patients' wishes
Chronic NSAID's users	Chronic NSAID's users
Before starting long‐term aspirin therapy	Before starting long‐term aspirin therapy
In patients with GERD requiring long‐term PPI	In patients with GERD requiring long‐term PPI
Gastric mucosal atrophy and or intestinal metaplasia	Gastric mucosal atrophy and or intestinal metaplasia

^a^ Addressed as indication in previous paediatric guidelines.

Adherence to the eradication regimen is the single most important reason causing failure of treatment when less than 90% of the prescribed drugs are taken. An additional cause of treatment failure is infection with multiple strains resistant to different antibiotics.

In conclusion, a high eradication rate diminishes the risk of re‐infection and reduces the spread of antibiotic resistance.

### 1.4 Benefits of Treatment of *H. pylori* in Children and Adults

The decision to treat must take into consideration the potential risk of developing complications later in life weighted against the potential risks of treatment. The benefits of treatment of *H. pylori* infection in children include prevention of gastric complications and associated non‐gastric complications as well as reduction of parental anxiety of nontreatment.

#### 1.4.1 Prevention of Gastric Complications

Gastric complications caused by *H. pylori* infection, including PUD, gastric atrophy/intestine metaplasia, gastric MALT lymphoma, and GC, can be prevented by eradication treatment. However, in children, the risk of PUD is low, and the risk of (pre)malignant complications is even lower. Unfortunately, currently, there are no good biomarkers that could help identify those children who will develop complications of infection later in life and who should receive eradication treatment also for preventive purposes.

Since *H. pylori* is the most common cause of gastric or duodenal erosions/ulceration in children and as the infection can be treated, guidelines suggest if erosions, ulcers, or scarring are visualized during upper endoscopy, to take biopsies to identify the presence of *H. pylori* . If the infection is confirmed, the eradication treatment is indicated [5].

It has also been shown that *H. pylori* infection is associated with the occurrence of premalignant lesions in children [42]. Infected children had a higher relative risk for antral and corpus chronic inflammation, activity, presence of lymphoid follicles, and, rarely, atrophy, whereas there was limited risk for intestinal metaplasia and only in the antrum [18]. Studies in adults have shown that with clearance of *H. pylori* infection, there is an improvement in mucosal atrophy and reduced development of intestinal metaplasia [43]. According to guidelines for adults, *H. pylori* should be eradicated when precancerous lesions are present [43]. As well, Japanese pediatric guidelines **r**ecommend eradication therapy for all *H. pylori* infected children in whom gastric atrophy (with or without intestinal metaplasia) is shown histologically [43].

Several studies investigated the effect of eradication therapy on the prevention of GC, and it has been shown in both high‐ and low‐prevalence settings that eradication of *H. pylori* infection is associated with a lower risk of GC than in persistent infection [21, 44, 45]. Recently, Ford et al. in their meta‐analyses of randomized controlled trials found that eradication of *H. pylori* in healthy asymptomatic individuals reduces future incidence of GC as well as GC‐related mortality [46].

#### 1.4.2 “Prevention” of Associated Non‐Gastric Complications

Previous meta‐analyses which included both adult and pediatric patients suggested an association between *H. pylori* infection and iron deficiency anemia. When a subgroup analysis considering different age groups was performed, for children and adolescents, a significant association between *H. pylori* and iron deficiency anemia was revealed. However, different diagnostic methods for diagnosing *H. pylori* infection contributed to the variation of the pooled estimates [47]. Nevertheless, it was also shown that *H. pylori* eradication therapy reduces the burden of anemia [48]. Recently published systematic reviews and meta‐analyses indicated that children with *H. pylori* infection are at a higher risk of developing anemia as compared to non‐infected children. The authors suggested that early diagnosis and treatment of *H. pylori* infection could play a crucial role in preventing anemia and improving overall health outcomes in affected children [49]. Therefore, pediatric guidelines suggest that if endoscopy is indicated after failure of therapy for iron deficiency anemia, testing for *H. pylori* may be considered and treated if found.

#### 1.4.3 Reduce Parental Anxiety of “Non‐Treatment”

Children with recurrent abdominal pain without any alarm signs or symptoms most likely have a disorder of gut‐brain interaction, independent of *H. pylori* status. Although not indicated, sometimes parents themselves perform non‐invasive tests for *H. pylori* and ask for treatment. Even if endoscopy is performed and if the infection is confirmed but no ulcers or erosions are found, *H. pylori* eradication has not been proven to improve symptoms. In such patients, according to ESPGHAN/NASPGHAN guidelines, the risks and benefits of treatment should be discussed with the parents and decided on treatment accordingly [5]. Hearing information on *H. pylori* infection and related risks from the doctor in addition to information widely available to the public can cause parental anxiety and make them more pro‐treatment oriented, even more so if they or their close family members have experienced diseases caused by *H. pylori* [50]. There are no such studies, but it is possible that sometimes the attempt to reduce parental anxiety might prevail in the decision to treat chronic *H. pylori* infection, especially due to the fear of parents and children for gastric carcinoma later in adulthood. However, diagnosis of *H. pylori* infection in children should be made according to the guidelines, and eradication treatment should be based on AST [5].

### 1.5 Treatment to Be Used in Adults and Children

One of the main differences in treatment between children and adults is that the “test and treat” strategy is not recommended for children. In childhood and adolescence, it is expected that treatment be given according to AST results tested during upper gastrointestinal endoscopy. In order to be in line with the principles of antibiotic stewardship strategies, it is important to avoid unnecessary drugs and therefore the best choice as a first‐line treatment remains a clarithromycin‐based triple therapy after confirmation of clarithromycin susceptibility, which can be done by cultivation or a real‐time PCR on gastric biopsies, or eventually on stool samples if an endoscopy cannot be performed (Figure 1) [51, 52]. The second antibiotic to be used is amoxicillin, which must be optimized regarding BMI (50 mg/kg) and frequency of administration (3–4 times a day) in agreement with the pharmacological properties of this drug. Finally, the choice of proton pump inhibitor (PPI) should be one that is not metabolized by the CYP‐2C19 liver enzyme, that is esomeprazole or rabeprazole and at high dosage.

In case it would not be possible to test clarithromycin susceptibility, a bismuth‐based quadruple therapy is the preferred empiric treatment as it leads to good results, although usually lower than the previous treatment and at the expense of more adverse events. Indeed, tetracycline resistance is very rare, and there is a limited impact of metronidazole resistance, which is one of the other drugs involved. There is no resistance to bismuth salts, and the PPI can be used at a normal dose. If bismuth salts are not available in the country, then triple therapy with PPI, amoxicillin, and metronidazole should be prescribed in children (Figure 1).

Treatment using clarithromycin without previous testing must be avoided, including concomitant treatment despite its success, because it leads to the administration of an unneeded antibiotic, which contributes to global bacterial resistance [53].

The conditions to use standard triple therapy are the same in adults and children. However, when a child is infected with clarithromycin‐resistant strain, a bismuth‐based quadruple therapy containing bismuth, PPI, amoxicillin, and metronidazole is indicated [5]. Tetracycline may be considered in bismuth‐based quadruple therapy only in children older than 8 years and with proven allergy to amoxicillin.

## 2 Conclusions

In the current review, we expose the differences in the effect of *H. pylori* infection between children and adults. Abovementioned differences led to the significant variations in the management guidelines and recommendations between the children and adults. In adults, eradication therapy is recommended to most infected patients. However, in children, the risk and benefits of eradication treatment described in this review must be considered when deciding “to treat or not to treat”. The minimal risks in childhood and the concerns related to treatment, including the more important negative effects of antibiotics at a young age, the risk of losing the possible “protective effect” of *H. pylori* , the possibility of treatment failure and re‐infection, cause restrictive treatment indications in children.

## Data Availability

The data that support the findings of this study are openly available.
